# 3D Patient-Specific Virtual Models for Presurgical Planning in Patients with Recto-Sigmoid Endometriosis Nodules: A Pilot Study

**DOI:** 10.3390/medicina58010086

**Published:** 2022-01-06

**Authors:** Giulia Borghese, Francesca Coppola, Diego Raimondo, Antonio Raffone, Antonio Travaglino, Barbara Bortolani, Silvia Lo Monaco, Laura Cercenelli, Manuela Maletta, Arrigo Cattabriga, Paolo Casadio, Antonio Mollo, Rita Golfieri, Roberto Paradisi, Emanuela Marcelli, Renato Seracchioli

**Affiliations:** 1 Division of Gynecology and Human Reproduction Physiopathology, Department of Medical and Surgical Sciences (DIMEC), Istituto di Ricovero e Cura a Carattere Scientifico (IRCCS), Azienda Ospedaliero-Univeristaria di Bologna, S. Orsola Hospital, University of Bologna, 40138 Bologna, Italy; giuliamaria.borghese@gmail.com (G.B.); manuela.maletta@studio.unibo.it (M.M.); paolo.casadio.unibo@gmail.com (P.C.); roberto.paradisi@unibo.it (R.P.); renat.seracchioli@gmail.com (R.S.); 2 Department of Radiology, Istituto di Ricovero e Cura a Carattere Scientifico (IRCCS) Azienda Ospedaliero-Universitaria di Bologna, 40138 Bologna, Italy; francesca.coppola@aosp.bo.it (F.C.); silvia.lomonaco@studio.unibo.it (S.L.M.); arrigo.cattabriga@studio.unibo.it (A.C.); rita.golfieri@unibo.it (R.G.); 3 Gynecology and Obstetrics Unit, Department of Neuroscience, Reproductive Sciences and Dentistry, School of Medicine, University of Naples Federico II, 80138 Naples, Italy; 4 Pathology Unit, Department of Advanced Biomedical Sciences, School of Medicine, University of Naples Federico II, 80138 Naples, Italy; antonio.travaglino.ap@gmail.com; 5 eDIMES Lab-Laboratory of Bioengineering, Department of Experimental, Diagnostic and Specialty Medicine (DIMES), University of Bologna, 40126 Bologna, Italy; barbara.bortolani@unibo.it (B.B.); laura.cercenelli@unibo.it (L.C.); emanuela.marcelli@unibo.it (E.M.); 6 Gynecology and Obstetrics Unit, Department of Medicine, Surgery and Dentistry “Schola Medica Salernitana”, University of Salerno, 84081 Baronissi, Italy; antmollo66@gmail.com

**Keywords:** three-dimensional image, anatomical models, endometriosis, magnetic resonance imaging, minimally invasive surgical procedures

## Abstract

*Background and Objective:* In recent years, 3D printing has been used to support surgical planning or to guide intraoperative procedures in various surgical specialties. An improvement in surgical planning for recto-sigmoid endometriosis (RSE) excision might reduce the high complication rate related to this challenging surgery. The aim of this study was to build novel presurgical 3D models of RSE nodules from magnetic resonance imaging (MRI) and compare them with intraoperative findings. *Materials and Methods:* A single-center, observational, prospective, cohort, pilot study was performed by enrolling consecutive symptomatic women scheduled for minimally invasive surgery for RSE between November 2019 and June 2020 at our institution. Preoperative MRI were used for building 3D models of RSE nodules and surrounding pelvic organs. 3D models were examined during multi-disciplinary preoperative planning, focusing especially on three domains: degree of bowel stenosis, nodule’s circumferential extension, and bowel angulation induced by the RSE nodule. After surgery, the surgeon was asked to subjectively evaluate the correlation of the 3D model with the intra-operative findings and to express his evaluation as “no correlation”, “low correlation”, or “high correlation” referring to the three described domains. *Results:* seven women were enrolled and 3D anatomical virtual models of RSE nodules and surrounding pelvic organs were generated. In all cases, surgeons reported a subjective “high correlation” with the surgical findings. *Conclusion:* Presurgical 3D models could be a feasible and useful tool to support surgical planning in women with recto-sigmoidal endometriotic involvement, appearing closely related to intraoperative findings.

## 1. Introduction

Endometriosis is a chronic inflammatory disease defined as the presence of functional endometrial tissue (stroma and glands) outside the uterine cavity, which has a tendency to invade and infiltrate [1]. The most severe form of the disease is deep infiltrating endometriosis (DIE), namely, the infiltration of retroperitoneal tissue or pelvic organs wall [2]. Bowel endometriosis occurs in 8% to 12% of women with the disease [3] and it is mainly represented by recto-sigmoid endometriosis (RSE) [4,5]. When indicated, surgery for RSE is challenging and may imply a high complication rate despite a comprehensive preoperative planning based on accurate medical history, clinical evaluation, and correct interpretation of imaging findings [6].

In recent years, the advances in diagnostic imaging and computer technology have led to a progressively wider use of 3D digital technologies and 3D printing to support surgical planning or to guide intraoperative procedures in various surgical specialties [7], including cardiovascular surgery [8,9], orthopedics [10], urology [11,12,13,14], maxillofacial surgery [15,16,17,18,19], and gynecology [20]. In particular, regarding gynecological surgery and endometriosis, Ajao M. et al. described the 3D printed model of a DIE nodule after laparoscopic removal [20].

On this basis, it would be conceivable that presurgical 3D models of DIE nodules, i.e., the 3D virtual anatomical model reconstruction of RSE nodules, could be a feasible and useful support for surgical planning in women with RSE. An improvement in surgical planning for RSE excision might help to further reduce the high complication rate related to this challenging surgery.

The aim of this pilot study was to build novel presurgical 3D models of RSE nodules, investigating the nodule’s circumferential extension, the bowel angulation, and the degree of bowel stenosis induced by the nodule, and to compare presurgical models with the intraoperative findings.

## 2. Materials and Methods

### 2.1. Study Protocol

The study was designed as an observational, prospective case series study according to an a priori study protocol. The study was reported according to the CARE Guidelines: Consensus-based Clinical Case Reporting Guideline Development. (CIT) [21].

Consecutive symptomatic women scheduled for minimally invasive surgery for RSE between November 2019 and June 2020 at our tertiary level center for endometriosis were enrolled. Patients with previous bowel or DIE surgery and/or younger than 18 years were excluded.

Preoperative MRIs (magnetic resonance imaging) from enrolled patients were used for building 3D models of RSE nodules and surrounding pelvic organs. Before surgery, the 3D virtual models were examined during multi-disciplinary preoperative planning, focusing especially on three domains: degree of bowel stenosis, nodule’s circumferential extension, and bowel angulation induced by the RSE nodule. Time between preoperative evaluation and surgery was recorded.

After surgery, the surgeon was asked to subjectively evaluate the correlation of the 3D model with the intra-operative findings and to express his evaluation as “no correlation”, “low correlation”, or “high correlation” referring to the three described domains.

### 2.2. Preoperative Evaluation

During preoperative work-up, as per usual clinical practice, all women underwent clinical and gynecological examination, transvaginal ultrasound scan performed by expert sonographers and MRI [22,23]. Demographic data were registered and pain symptoms severity (chronic pelvic pain, dysmenorrhea, dyspareunia, dysuria, and dyschezia) was assessed through the 11-point Visual Analogue Scale (VAS) [24]. Endometriosis stage was defined using the revised classification of the American Society for Reproductive Medicine (rASRM) [25].

### 2.3. MRI Acquisition

Bowel preparation implied 2 days of low-fiber diet and oral administration of Macrogol-Na-K 140 gr the day before the imaging. Before the examination, rectal opacification with at least 150 mL of ultrasound gel solution was required to better evaluate the degree of bowel stenosis. An antispasmodic agent (scopolamine-N-Botyl bromide) was administered intravenously immediately before the exam to reduce bowel peristalsis and the artifacts due to the bowel movement. Women lay in prone position and with moderate urinary bladder repletion for the optimal visualization of both the recto-sigmoid tract and the excretory system on the urography phase.

In our institution, MRI is performed with a 1.5 T superconducting system (GE Healthcare, Chicago, IL, USA). The MRI protocol for women with pelvic endometriosis includes a 3-plane single shot fast spin echo (SS-FSE) sequence as well as localization sequences to identify the longitudinal axis of the uterus. Subsequently, high resolution (3 mm thickness) sagittal T2-weighted FSE sequences oriented towards the longitudinal axis of the uterus and high resolution (3 mm thickness) oblique axial T2-weighted FSE sequences oriented perpendicularly to the longitudinal axis of the uterus are performed. Axial plane enhanced Diffusion Weighted Imaging (eDWI) sequences with B value of 0, 600, and 1000 are acquired. Before intravenous administration of the contrast agent, fat suppressed T1-weighted 3D LAVA FLEX on axial plane and fat suppressed T1-weighted 3D LAVA XV in oblique coronal plane sequences parallel to the longitudinal axis of the uterus are acquired.

After intravenous injection of paramagnetic contrast medium (Gadoteridiol 0.2 mL/kg), a dynamic study with fat suppressed T1-weighted 3D LAVA XV on oblique coronal plane at 40 s e 70 s is carried out. As a tardive venous phase (about 3 min after the contrast injection) a T1-weighted 3D LAVA FLEX sequence on axial and sagittal plane is acquired, as well as a T1-weighted 3D LAVA XV sequence on coronal plane for the urographic phase 10 min after the contrast injection.

Menses was not a contraindication for MRI study.

### 2.4. 3D Modelling

All 3D virtual anatomical reconstructions based on preoperative MRI were carried out at the eDIMES Lab of the S. Orsola-Malpighi Hospital, University of Bologna, Italy, by radiologists and engineers working in continuous interaction with surgeons. The segmentation, i.e., the labelling of each structure of interest in each MRI slice was obtained using D2P^TM^ software (‘DICOM to PRINT’; 3D Systems Inc., Rock Hill, SC, USA), a modular software package designed to create 3D digital anatomical models from medical imaging data and certified for using the virtual models in surgical planning, advanced visualization, and 3D printing. D2P™ consolidates all 3D model preparation steps into a single workstation, while relying on unique automatic segmentation tools and functions dedicated to specific anatomical structures, such as vessels, solid organs, thin bones, teeth, and mandible and maxilla bone structures. The segmentation process itself was carried out by a radiologist with more than 10 years of experience on pelvic MRI imaging, and previous experience in segmentation on different software, assisted by two resident doctors and working in close cooperation with biomedical engineers.

To obtain the segments, sagittal T2-weighted images oriented towards the longitudinal axis of the uterus and high-resolution oblique axial T2-weighted images oriented perpendicularly to the longitudinal axis of the uterus were contoured. Standard MRI sequences for the pelvis study were useful to better identify the lesion and its anatomical relationships with the adjacent organs. In some cases, coronal T1-weighted 3D LAVA XV images, acquired during the urographic phase, were processed in order to reconstruct the ureteral course.

Semi-automatic tools (multi-slice interpolation and threshold segmentation) of D2P^TM^ software were used to segment each anatomical structure of interest (i.e., the endometriotic nodule and surrounding organs including rectum, uterus, vagina, and urinary bladder).

**Figure 1:**
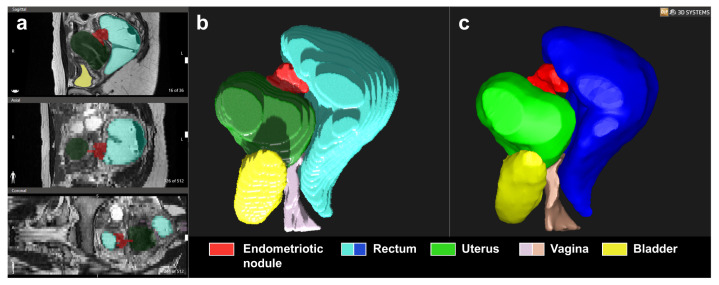
Display of the process of obtaining the 3D virtual anatomical model (**c**) by segmentation of the anatomical regions of interest (**b**) based on the magnetic resonance imaging (MRI) scan (**a**), using D2P^TM^ software (3D Systems).

D2P^TM^ was also used to convert the segmented two-dimensional structures into 3D triangulated surface mesh file (STL), using mesh creation methods of D2P^TM^ (contour or gridbase). A display of the segmentation and 3D modelling outcomes is reported in Figure 1.

### 2.5. Surgical Technique

All surgical procedures were performed by the same surgeons, highly experienced in minimally invasive surgery for endometriosis. The abdominopelvic cavity was examined to exclude the presence of endometriotic lesions. Adhesiolysis, the excision of superficial endometriosis implants, ovarian cystectomy through stripping technique, and temporary ovarian suspension, when required, were performed before approaching DIE as per usual clinical practice. In case of ureteral endometriosis or ureteral involvement by surrounding fibrosis, conservative or radical procedures were performed, as previously reported [2,26]. RSE was removed using a conservative (shaving) or radical (segmental or discoid resection) approach as previously described [27]. Shaving technique was attempted first in all patients. In case of persistence of stenosis of the bowel lumen, deep infiltration through the bowel wall, or severe damage of the muscularis propria, we subsequently proceeded to either a discoid anterior rectal wall resection or a segmental resection. If the implants were < 3 cm in maximum diameter and within 15 mm from the anal verge, we performed a discoid resection, if the nodules were > 3 cm or > 15 cm from the anal verge, segmental resection was chosen. All specimens were sent for routine histological examination. Endometriosis was considered histologically confirmed when endometrial glands and stroma were found in the examined specimens.

### 2.6. Ethical Statement

The study was carried out in accordance to the Helsinki Declaration and received approval by the Institutional review Board of the University of Bologna, Italy (no: 115/2021/Oss/AOUBo).

All women signed a written informed consent for enrollment in the study and for the images to be published. Patients’ records and data were anonymized before the 3D model development.

## 3. Results

### 3.1. Study Population and 3D Virtual Models

During the study period, 7 women meeting the selection criteria were enrolled.

Demographic, preoperative, and surgical data of the enrolled women are summarized in Table 1.

All the included women underwent surgery within 2 months from the preoperative multi-disciplinary planning. Pathological examination confirmed the endometriotic histology of all the removed nodules.

Three-dimensional rendering and views of the reconstructed anatomical virtual models of RSE nodules and surrounding pelvic organs generated from MRI and their comparison with 2D-MRI views were obtained for all enrolled patients. The 3D virtual models can be rotated and zoomed and are navigable, i.e., the surgeon can interact with them by changing the transparency of each structure in the 3D rendering, as well as creating a detailed view of the interaction among the different anatomical structures (Supplementary Video S1).

### 3.2. Surgeons’ Judgements

Before surgery, the 3D virtual models of RSE nodules were examined during multi-disciplinary preoperative planning, focusing especially on three domains: degree of bowel stenosis, nodule’s circumferential extension, and bowel angulation induced by the RSE nodule. After surgery, the surgeon was asked to subjectively evaluate the correlation of the 3D model with the intra-operative findings and to express his evaluation as “no correlation”, “low correlation”, or “high correlation” referring to the three described domains.

In all cases, surgeons reported a subjective “high correlation” between the 3D anatomical virtual model and the surgical finding of the RSE nodule.

**Figure 2:**
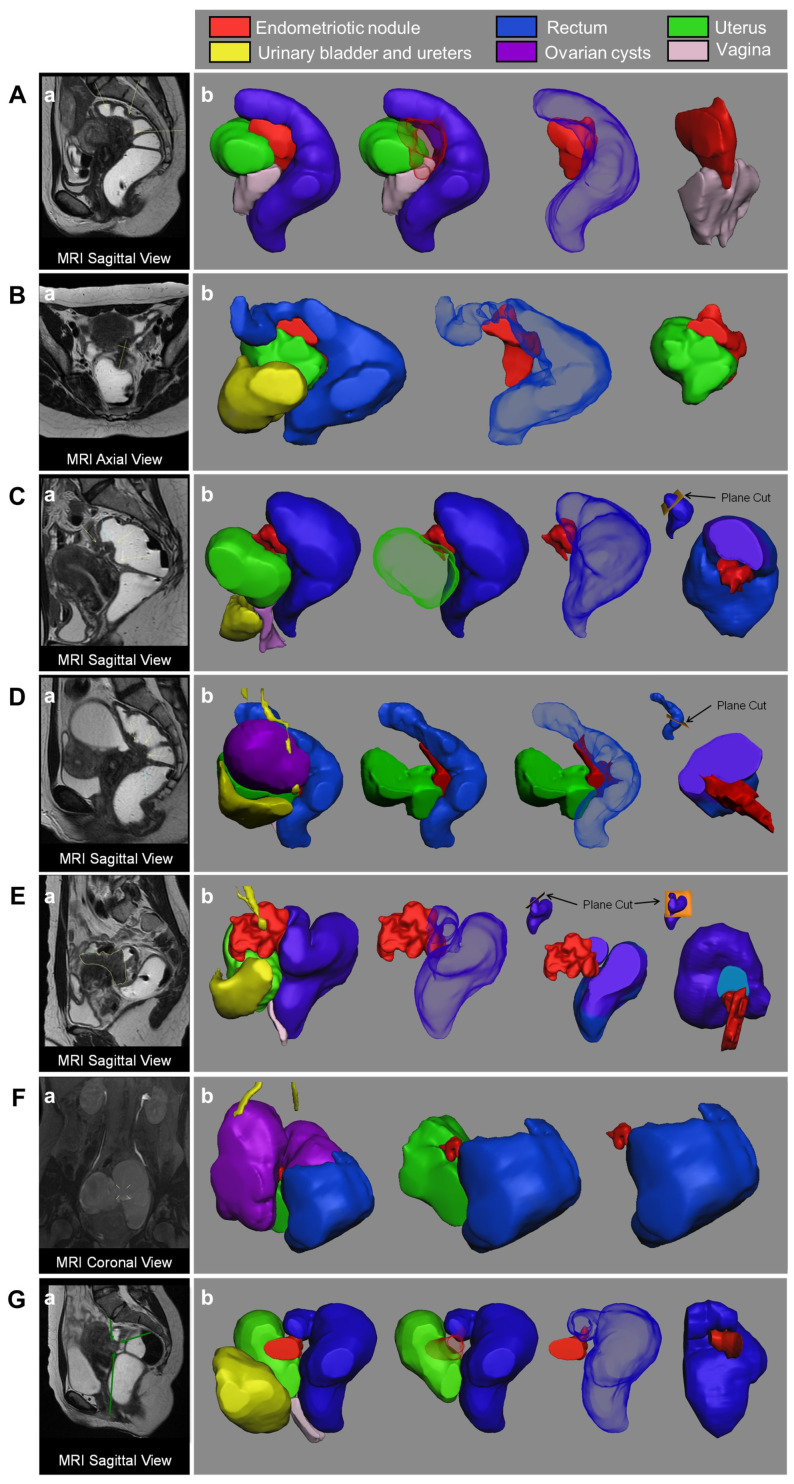
Two-dimensional MRI images (a) and 3D models views (b) of the 7 included women. The 3D anatomic reconstruction of the recto-sigmoid endometriosis (RSE) nodule with the surrounding pelvic organs is presented, demonstrating their spatial relationship, as well as the isolated nodule to show the nodule’s extension (**A**–**G**). The cutting plane is shown in cases C, D and E.

**Figure 3:**
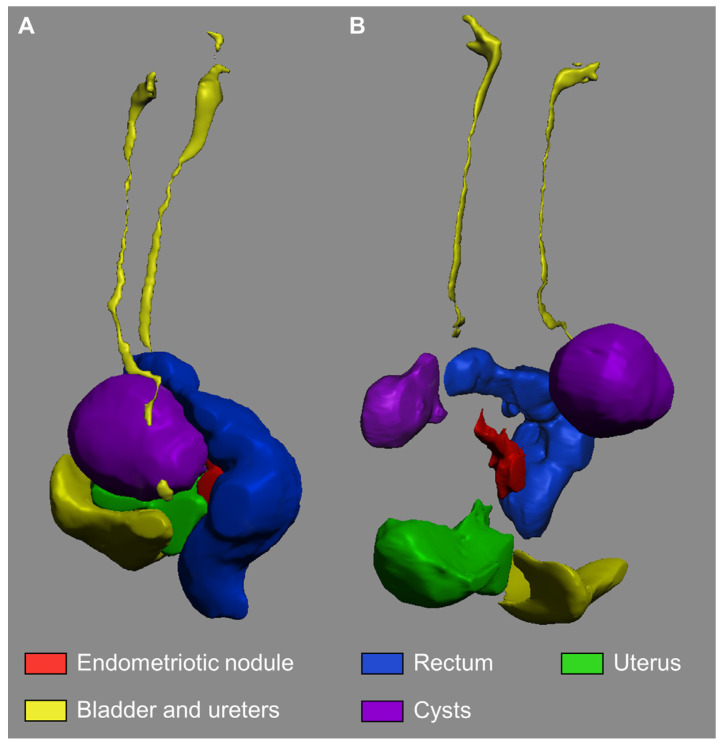
Display of case D 3D anatomic model including ureteral course (**A**). An exploded view of the anatomical relationship is presented (**B**).

Figure 2 and Figure 3 report 2D MRI images and a collection of views of the 3D models of the seven included cases.

## 4. Discussion

This pilot study shows a subjective high correlation between the 3D anatomical virtual model and the surgical finding of the RSE nodule, with particular regard to degree of bowel stenosis, nodule’s circumferential extension and bowel angulation induced by the RSE nodule. In the future, presurgical 3D models could be a feasible and useful tool to support surgical planning in women with recto-sigmoidal endometriotic involvement.

RSE represents about 90% of all intestinal lesions [6,28], and it manifests mainly with intestinal symptoms such as constipation, dyschezia, and rectal bleeding [29], as well as dysmenorrhea, chronic pelvic pain, dyspareunia, and infertility [30,31]. In cases of symptomatic women with contraindications or poor response to medical treatment or presenting with bowel occlusive symptoms, hydroureteronephrosis, or infertility not responding to assisted reproductive technologies, surgery is indicated [6]. Although bowel surgery for endometriosis was proven to improve both symptoms and quality of life [32,33], the surgical approach is often challenging and can imply a high complication rate [6]. Perioperative complications include hemorrhage, need for laparotomic conversion, infections, and anastomotic leaks (especially in case of low rectal resection), while the main postoperative complains are fistulas and urinary or bowel disfunctions, especially constipation [3,6,30].

A detailed and thorough preoperative anatomical and morphological study based on accurate imaging techniques appears therefore mandatory in order to reduce potential complications. Transvaginal ultrasound scan and MRI were proven to be accurate diagnostic tools for DIE [34,35]. According to a recent Cochrane review, MRI showed 94% sensitivity and 77% specificity for DIE detection, similar to those shown by transvaginal ultrasound scan. In particular, MRI sensitivity and specificity for RSE mapping were 92% and 96%, respectively [36]. Bousard et al. demonstrated the high sensitivity of MRI in identifying muscular layer infiltration by bowel endometriosis, especially of the “fan sign” (sensibility 100%, specificity 75%) and of the “mushroom cap sign” on conventional T2-weighted images [37]. Proper surgical planning is based on correct interpretation of the imaging findings, as well as on accurate medical history and clinical evaluation. Nevertheless, a complete understanding of distorted anatomical landmarks is often difficult to obtain [38]. Indeed, surgeons should mentally reconstruct complex 3D anatomical structures basing on 2D images; this process could be particularly tough for novices, residents, and junior surgeons. In several surgical settings, the introduction of 3D modelling provided many advantages, such as deeper understanding of anatomy, more affordable preoperative planning, and better interaction within the multidisciplinary team and prompted alternative operative scenarios [39]. Furthermore, it offered wide and specific didactic opportunities. In fact, 3D printing, i.e., the translation of the reconstructed 3D virtual model into a physical object, may offer a practical hands-on approach with not only visual but also tactile feedback [38]. Delivery time and production costs still limit the widespread diffusion of 3D printing in the routine clinical setting. According to Malik H. et al., main costs are due to hardware, and software, which could account for up to USD 40,000 [40]. The available literature reports that the overall time to complete the printing process, from image elaboration to the final output ranges between about half a day to several weeks [40,41]. At this stage, 3D printing is therefore suitable only for elective surgery [41]. Minor drawbacks include the inability to reproduce extremely small or thin structures due both to the limitations imposed by most printing materials and to the resolution of current cross-sectional scanners for computed tomography (CT) or MRI [41].

In gynecology, preliminary experiences on the applications of 3D modelling are reported. Three-dimensional reconstruction was used for didactic purposes to provide realistic models for perineal repair and anal sphincter sutures and for cervical cancer recognition and diagnosis [42,43,44], as well as in the field of infertility and assisted reproductive technologies [45]. Three-dimensional modelling of female bone pelvis was employed to identify risk factors for pelvic floor disorders study and for dystocia [46,47]. Rubod C. et al. investigated the physiology of the pelvic mobility and the mechanism of genital prolapse through a 3D reconstruction [48]. Han Y. et al. used 3D modelling to investigate the topographic relationship between ureters and iliac vessels bifurcation in 129 women undergoing CT angiography and CT urography [49]. Li J. et al. [50] and Duan H. et al. [51] described iliac vessels distribution based on 3D reconstruction, in order to reduce the risk of vessel injury during pelvic surgery, especially lymphadenectomy [50,51]. Some authors used 3D modelling for the preoperative planning of complex surgical cases. Mackey A. et al. reported the MRI-based 3D reconstruction of a uterine model to be used in the multidisciplinary preoperative planning for a woman with multiples myomas scheduled for cesarean section [52]. MRI-based 3D printing of polyol-isocyanate models of uterus and iliac vessels was described in five patients diagnosed with endometrial cancer and scheduled for hysterectomy; its usefulness was positively evaluated by radiologists, surgeons, and patients [53]. Baek M. et al. developed a 3D model of uterus, cervix, and cancer of a patient with locally advanced cervical cancer undergoing radical hysterectomy [54]. A presurgical 3D reconstruction of a complex female genital tract malformation was also described [55].

Regarding the endometriosis field, only one case report from Ajao M. et al. is described in the literature [20]. The authors retrospectively compared a 3D printed model of a DIE nodule with its intraoperative features and showed a close correlation of the nodule-uterine wall-rectum model with the real scenario, especially regarding the spatial relationship, suggesting a potential preoperative role of 3D models of DIE in surgical planning.

Based on this experience, we hypothesized that 3D modelling could be a feasible and useful support for surgical planning in women with recto-sigmoidal endometriotic involvement. We therefore reconstructed a 3D model of the posterior endometriotic nodule and surrounding organs in seven patients with RSE.

According to the surgeons, 3D representations of the RSE nodules were closely related to intraoperative findings. In particular, the surgeons found the visualization of the three-dimensional anatomical reconstruction before surgery to be a useful support for the evaluation of nodule’s circumferential extension and of the bowel angulation induced by the RSE implant. Furthermore, the degree of bowel stenosis is of paramount importance in choosing the proper surgical approach for bowel lesions and a cut-off is not well established—recently, cut-off of 30% bowel stenosis has been proposed as a risk factor for colorectal resection [56]. When the normal intestinal caliber is not restored after the conservative approach (rectal shaving), bowel resection is warranted. The decision to proceed with bowel resection, and the choice between anterior discoid rectal wall resection (for implants <3 cm on the rectum anterior surface within 15 cm from the anal verge) and segmental resection, is mainly based on the intraoperative subjective surgeon’s evaluation. In this series, the surgical approach to bowel lesion (rectal shaving versus discoidal anterior or segmental resection) hypothesized after 3D model analysis was confirmed at the intraoperative evaluation. Moreover, clinicians reported that the degree of bowel stenosis shown by the 3D model was helpful in discussing with the women the surgical plan and the possible need for a bowel resection.

Based on this preliminary experience, we consider that 3D modelling of endometriotic nodules could improve surgeons’ ability to obtain and use the information from MRI during surgical planning, facilitating the mental process of 3D reconstruction of pelvic structures from bidimensional images. In detail, we think that the main information provided by this technique are nodule’s circumferential extension, bowel angulation induced by the RSE implant, and degree of bowel stenosis. The availability of these data could play an important role in preoperative planning in a multidisciplinary team. Furthermore, we consider that 3D models offer didactic opportunities on different levels. Indeed, junior, or less experienced surgeons, could benefit from a deeper understanding of the anatomical landmarks both during preoperative workup and during surgical procedures (for pelvic orientation). On the other hand, as a limitation, the 3D models did not allow the ureteral course to be fully reconstructed, mainly due to ureteral peristalsis. Moreover, in future studies, it would also be interesting to investigate 3D modelling’s possible role in complex anatomical settings, such us in women who have previously undergone bowel surgery.

Despite the novelty of our findings, the small sample size limits the impact of our study. Furthermore, as another limitation, the 3D images were reviewed in a preoperative multi-disciplinary planning as well as 2D MRI images, possibly leading to an interpretation bias. In future studies, our findings could be strengthened by the evaluation of inter- and intra-observer agreement of different surgeons about the correlation between 3D models and surgical anatomical scenario. Moreover, the information provided by the 3D reconstructed models should be compared with those obtained from other diagnostic tools such as transvaginal ultrasound and MRI.

## 5. Conclusions

The presurgical 3D virtual anatomical model reconstruction could be a feasible and useful tool to support surgical planning in women with recto-sigmoidal endometriotic involvement. Three-dimensional models appeared closely related to intraoperative findings, especially referring to nodule’s circumferential extension, bowel angulation induced by the RSE implant, and degree of bowel stenosis. In the future, 3D modelling could also provide a novel didactic opportunity for trainees and junior surgeons. Further studies are advocated to confirm and strengthen our preliminary findings, to evaluate 3D modelling effectiveness and possible role in optimizing surgical performance and reducing surgical complications.

## Figures and Tables

**Table 1:** Demographic, preoperative, and surgical data of the enrolled women.

Baseline Characteristics	
Age, mean ± SD ^a^, years	35 ± 3
BMI, mean ± SD ^a^, kg/m²	22.3 ± 2.4
Parity ≥ 1, *n* ^b^ (%)	0/7 (0.0%)
Seek pregnancy, *n* (%)	2/7 (28.6%)
Preoperative estrogen progestin combination therapy, *n* (%)	2/7 (28.6%)
Preoperative progestin therapy, *n* (%)	4/7 (57.1%)
Previous surgery for endometriosis, *n* (%)	3/7 (42.9%)
Preoperative symptoms	
Dysmenorrhea *n* (%)	6/7 (85.7%)
Chronic Pelvic Pain *n* (%)	3/7 (42.9%)
Periovulatory pain *n* (%)	2/7 (28.6%)
Dyschezia *n* (%)	3/7 (42.9%)
Hematochezia *n* (%)	1/7 (14.3%)
Constipation *n* (%)	3/7 (42.9%)
Diarrhea *n* (%)	2/7 (28.6%)
Dyspareunia *n* (%)	3/7 (42.9%)
Dysuria *n* (%)	0/7 (0.0%)
MRI ^c^ findings	
Uterine adenomyosis *n* (%)	2/7 (28.6%)
Endometrioma *n* (%)	3/7 (42.9%)
Bilateral endometrioma *n* (%)	2/7 (28.6%)
Intestinal endometriosis *n* (%)	7/7 (100.0%)
Recto-sigmoidal endometriosis *n* (%)	7/7 (100.0%)
Maximum diameter of posterior endometriosis nodule, mean ± SD, mm	42.9 ± 17.7
Bowel stenosis *n* (%)	5/7 (71.4%)
Vagina endometriosis, *n* (%)	2/7 (28.6%)
Parametrium endometriosis, *n* (%)	2/7 (28.6%)
Endometriosis of the utero-sacral ligaments, *n* (%)	5/7 (71.4%)
Hydronephrosis *n* (%)	0/7 (0.0%)
Surgical data	
Hysterectomy, *n* (%)	0/7 (0.0%)
Monolateral salpingectomy, *n* (%)	1/7 (14.3%)
Bilateral salpingectomy, *n* (%)	0/7 (0.0%)
Ovariectomy, *n* (%)	0/7 (0.0%)
Ovarian cystectomy, *n* (%)	4/7 (57.1%)
Monolateral, *n* (%)	2/7 (28.6%)
Bilateral, *n* (%)	2/7 (28.6%)
Bladder shaving, *n* (%)	0/7 (0.0%)
Cystectomy, *n* (%)	0/7 (0.0%)
Rectal shaving, *n* (%)	3/7 (42.9%)
Anterior discoid bowel resection, *n* (%)	0/7 (0.0%)
Segmental bowel resection, *n* (%)	4/7 (57.1%)
Low bowel resection, *n* (%)	1/7 (14.2%)
Distance anus, mean ± SD, cm	6.8 ± 3.7
Appendectomy, *n* (%)	0/7 (0.0%)
Monolateral ureterolysis, *n* (%)	3/7 (42.9%)
Bilateral ureterolysis, *n* (%)	1/7 (14.3%)
Ureteral nodule removal, (%)	0/7 (0.0%)
Ureterectomy, *n* (%)	0/7 (0.0%)
Vaginal opening, *n* (%)	3/7 (42.9%)
Surgery duration, mean ± SD, min	198 ± 51

^a^ Standard Deviation; ^b^ Number of women; ^c^ Magnetic Resonance Imaging.

## Data Availability

Data are available on request due to restrictions, e.g., privacy or ethical.

## Supplementary Materials

The following are available online at https://www.mdpi.com/article/10.3390/medicina58010086/s1, Video S1: Three-dimensional rendering of the reconstructed anatomical model of a recto-sigmoidal endometriosis nodule and the surrounding pelvic organs.
